# Pulmonary Alveolar Microlithiasis: An Isolated Case in a Hispanic Male

**DOI:** 10.1155/2020/6247920

**Published:** 2020-03-27

**Authors:** Preethi Dileep Menon, Sarah Hackman

**Affiliations:** Department of Pathology and Laboratory Medicine, University of Texas Health, 7703 Floyd Curl Drive, MC#7750, San Antonio, Texas 78229, USA

## Abstract

Pulmonary alveolar microlithiasis (PAM) is an uncommon hereditary lung disease characterized by widespread deposition of calcium phosphate microliths within the alveolar spaces. It is considered an autosomal recessive disease with a mutation in a gene encoding a sodium phosphate cotransporter. The imaging findings in the early phase of disease can be mistaken for miliary tuberculosis or sarcoidosis. However, the classic radiologic findings in the later phases of disease show numerous opacities causing a “snowstorm” appearance to the lungs that corresponds with widespread deposition of microliths throughout the lung parenchyma. Although the disease often progresses over a slow time course, there are no effective therapies, and bilateral lung transplantation is recommended when there are increasing oxygen requirements or evidence of pulmonary hypertension.

## 1. Introduction

Pulmonary alveolar microlithiasis (PAM) is an uncommon hereditary lung disease characterized by widespread deposition of calcium phosphate microliths within the alveolar spaces. It is considered an autosomal recessive disease with a mutation in a gene that encodes a sodium phosphate cotransporter. PAM can be diagnosed with classic imaging findings of numerous calcifications in the advanced phases of the disease. Although often occurring in families and more common in Japan, Turkey, and Italy, here we report an isolated case of a Hispanic young adult with classic radiographic and histologic findings.

## 2. Case Report

**Figure 1:**
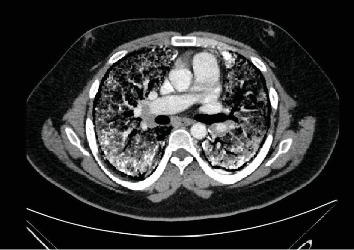
Chest CT scan with diffuse ground glass opacities and punctate calcifications involving bilateral lungs.

A 24-year-old Hispanic male presented to outpatient clinics with a past medical history of a persistent cough and shortness of breath for 10 years. Recently, his symptoms progressively worsened to chronic hypoxemic respiratory failure. He required long-term supplemental oxygen therapy of 5 liters/minute by nasal cannula and high flows of up to 15 liters/minute during pulmonary rehabilitation. His pulmonary function tests revealed a markedly decreased forced vital capacity (FVC) and diffusing capacity of the lung for carbon monoxide (DLCO), which were only 35% and 34% of predicted, respectively. Computed tomography (CT) of the chest revealed diffuse ground glass opacities, punctate calcifications, and extensive subpleural cysts consistent with PAM (Figure 1). A prominent pulmonary trunk, right cardiac chamber dilation, and mild interventricular septum straightening suggested a component of pulmonary artery hypertension. Right heart catheterization confirmed pulmonary artery hypertension with normal cardiac output and cardiac index. Because of his progressive symptoms with significant oxygen requirements, he underwent bilateral lung transplantation.

**Figure 2:**
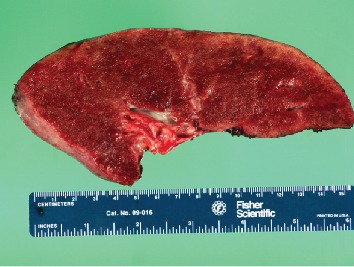
Gross photograph of explanted lung with a gritty, sand-like texture.

**Figure 3:**
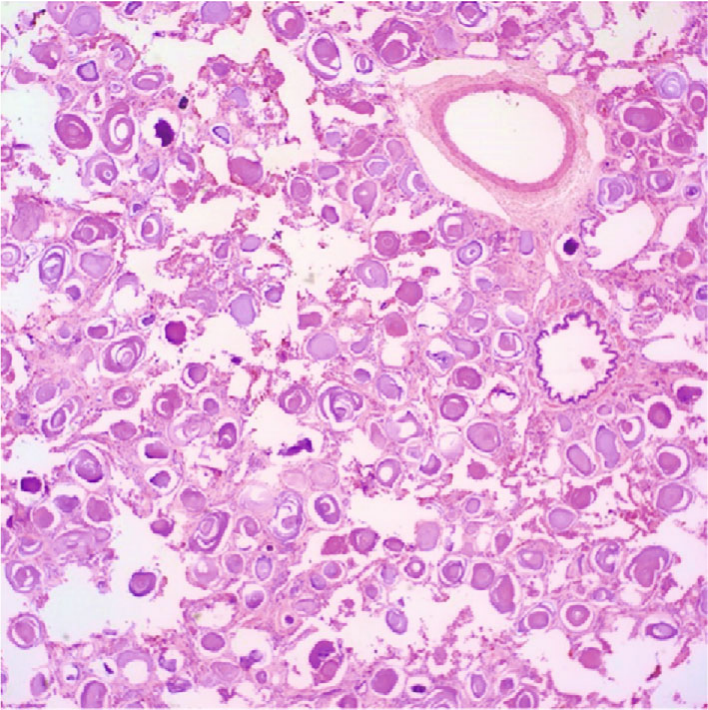
Hematoxylin- and eosin-stained sections with diffuse intra-alveolar deposition of lamellar spherical microliths, 40x.

Gross examination of the bilateral pneumonectomy specimens revealed enlarged lungs (right lung 1750 grams, left lung 1438 grams). The pink-tan pleural surfaces had multiple small plaque-like structures. The cut surfaces were pink-red and spongy with a gritty, sand-like texture (Figure 2). Hematoxylin- and eosin-stained sections revealed the characteristic diffuse intra-alveolar lamellar microliths (Figure 3). The background lung parenchyma showed interstitial fibrosis, numerous hemosiderin-laden macrophages, interstitial inflammation, and acute pleuritis.

His lung transplantation was complicated by intraoperative hemorrhage requiring >20 units of red cells and various other blood products. His hospital course was complicated by persistent leukocytosis and stenosis to the left mainstem bronchus, mucosal ischemia, and recurrent mucous plugging requiring repeated bronchoscopies. At 1 year post transplant, his pulmonary function tests revealed an improved FVC and DLCO of 50% and 76% of predicted, respectively. Since PAM has an autosomal pattern of inheritance, family screening was being considered for this patient.

## 3. Discussion

PAM is a hereditary lung disease with an autosomal recessive pattern of inheritance. The first detailed macroscopic description of this entity occurred in 1686, but it was centuries later that the gene responsible for the condition, solute carrier family 34 member 2 (SLC34A2), was described. Although normally expressed in several human tissues of epithelial origin, mutations in SLC34A2 produce a defective sodium phosphate-IIb transporter protein. As a result, alveolar epithelial type II cells are not able to clear phosphorus ions, which leads to calcium phosphate deposits and microlith formation in the extracellular fluid [1]. The microliths appear smaller, but similar to corpora amylacea [2]. Approximately 1000 cases of PAM are reported worldwide with the majority occurring in Asia and Europe [3]. Turkey had the highest incidence and was followed by China, Japan, and India [3–5]. Approximately 50 cases have been reported in the USA [3]. PAM is not associated with a significant sex predilection and is described at all ages, more frequently in the second and third decades of life [3–5]. Castellana et al. also revealed that the majority of PAM cases showed homozygous mutations in the SLC34A2 gene [3, 6]. Additionally, the higher prevalence of PAM in certain areas is attributed to the high proportions of consanguinity rather than founder effect [3].

PAM has been subdivided into four evolutionary phases based on radiology, which begins in the precalcific stage, through diffusely scattered calcifications, to greater numbers of calcifications with ground glass change and interstitial thickening and finally to a near “white out” of lung parenchyma on imaging [7, 8]. The second and third phases show characteristic imaging findings of “sandstorm” and “crazy paving,” respectively. These later phases are often sufficient to make a diagnosis of PAM [8].

Early imaging with only small numbers of the microliths raises pulmonary tuberculosis, sarcoidosis, or hemosiderosis into the differential diagnoses since all of these conditions can present with diffuse opacifications as well as alveolar proteinosis or pulmonary metastatic calcification [9]. Since the prevalence of PAM seems to be more frequent in countries where tuberculosis is common, there is usually a higher degree of suspicion for tuberculosis, due to the rarity of PAM and similar imaging features. In one review, PAM was incorrectly diagnosed as miliary tuberculosis in >72 cases [3]. While the majority of the differential diagnoses present with a more severe clinical course, the clinical course of PAM is variable, ranging from slowly progressive disease to a rapid onset and may eventually lead to cor pulmonale or respiratory failure [3]. Pulmonary tuberculosis presents with cough, weight loss, fatigue, and fever that is often associated with a chronic, debilitating disease or immunosuppressive states with microscopic examination revealing granulomatous inflammation that may progress to fibrosis and calcification. Sarcoidosis may be asymptomatic or present with symptoms similar to pulmonary tuberculosis and often shows multiple nonnecrotizing interstitial epithelioid granulomas. Alveolar proteinosis presents with fever, cough, dyspnea, and chest pain with microscopic examination revealing periodic acid-Schiff-positive proteinaceous material filling the alveoli with preservation of normal alveolar architecture. Pulmonary metastatic calcification can occur in any age group in response to various conditions such as epithelial and lymphoproliferative malignancies, posttransplant and renal failure with microscopic examination showing a haphazard distribution of calcified material in the lung parenchyma. Although imaging, particularly during the early/precalcific phases of the disease, could raise the possibility of the abovementioned differential diagnoses, these entities can usually be excluded based on a careful review of symptoms and histological assessment.

If the imaging findings are not diagnostic for PAM, a tissue diagnosis may be required. As seen in our patient's case, alveolar spaces contain abundant microliths or intra-alveolar spherical calcifications that range from 50 to 1000 *μ*m in diameter [10]. These microliths are predominantly composed of calcium and phosphorus and are periodic acid-Schiff-positive [11]. When microliths are present in abundance, the histology is unmistakable. A bronchoalveolar lavage can reveal microliths with fewer complications than transbronchial biopsy or open lung biopsy. An accurate diagnosis of PAM is important because it can carry implications for symptoms at extrapulmonary sites. Because the SLC34A2 gene is widely expressed, there have been reports of microlith deposition within seminal vesicles or periurethral tissue with testicular atrophy or azoospermia as a result [3].

A variety of invasive and noninvasive modalities including calcium chelating agents, systemic corticosteroids, and serial bronchopulmonary lavage are described as palliative treatments [12–15]. The use of diphosphonates was first introduced by Göcmen et al. [16]; however, subsequent clinical trials demonstrated little to no benefit [17, 18]. Ozcelik et al. published 2 cases of PAM that were treated with diphosphonates for 9 and 11 years, respectively, with a beneficial response [19]. They supposed that factors such as the onset of initial treatment, duration, and the dosage of the medicine could influence the result of the treatment. There is no known medical or gene therapy capable of reducing the progression of the disease. At present, lung transplantation remains the only possible treatment for end-stage disease when either right heart failure or severe respiratory failure is present. Bilateral lung transplantation is preferred to avoid persistent shunting of blood to the native lung [20].

## 4. Conclusion

PAM is an uncommon hereditary lung disease with approximately 50 cases reported in the USA and is characterized by widespread deposition of calcium phosphate microliths within the alveolar spaces. While the diagnosis can be accurately made with classic imaging findings, there are no guidelines for the treatment of PAM. Lung transplantation is the only effective treatment for this disease.
